# Amygdala reactivity to social stress in young adults: A scoping review of neuroimaging studies

**DOI:** 10.1016/j.ynstr.2026.100805

**Published:** 2026-03-21

**Authors:** Maribelle Nassar, Hayat Harati, Elias Estephan, Elie Al Ahmar, Lea Saab, Mariebelle Saab

**Affiliations:** a Neuroscience Research Center, Faculty of Medical Sciences, Lebanese University, P.O. Box 6573/14, Beirut, Lebanon; b Faculty of Engineering, Sagesse University, Furn El Chebbak, P.O. Box 50501, Baabda, Lebanon; c LBN, University of Montpellier, Montpellier, 34193, France; d Faculty of Pedagogy, Lebanese University, Furn El Chebbak, Beirut, Lebanon

**Keywords:** Amygdala, Stress, Adulthood, Neuroimaging, fMRI, Connectivity

## Abstract

**Introduction:**

Social stress substantially influences emotional and cognitive functioning and is a key contributor to vulnerability for stress-related psychopathology in young adults. The amygdala plays a central role in mediating neural responses to socially evaluative stressors. Although prior neuroimaging studies have examined amygdala reactivity under social stress, findings remain heterogeneous, partly due to individual and methodological variability. Clarifying these patterns is essential for advancing neurobiological models of stress vulnerability.

**Methods:**

Following PRISMA-ScR guidelines, PubMed, ScienceDirect, Google Scholar, and BioRxiv were searched for studies published between 2018 and 2024. Eligible studies included healthy participants aged 17–35 and employed validated social stress paradigms. Functional magnetic resonance imaging (fMRI) studies formed the primary focus, with electroencephalography (EEG) studies included when they provided complementary temporal insights into stress-related neural dynamics. Data extraction captured key study characteristics, imaging approaches, stress paradigms, and individual difference variables.

**Results:**

Thirteen studies met inclusion criteria. Most fMRI studies reported increased amygdala reactivity during socially evaluative stress. Functional and effective connectivity analyses frequently demonstrated reduced coupling between the amygdala and prefrontal regulatory regions, consistent with impaired top-down emotional control. Individual factors, including sex, genetic variation, resilience, and neurochemical profiles, contributed to variability in neural responses. EEG findings provided complementary evidence of stress-related alterations in oscillatory dynamics associated with threat processing and regulatory processes.

**Conclusion:**

Social stress in young adults is commonly associated with heightened amygdala reactivity and altered amygdala-prefrontal connectivity, with substantial interindividual variability. These findings support a shift toward more individualized neurobiological models of stress processing and highlight the importance of methodological standardization and multimodal neuroimaging in future research.

## Graphical abstract

**Figure:**
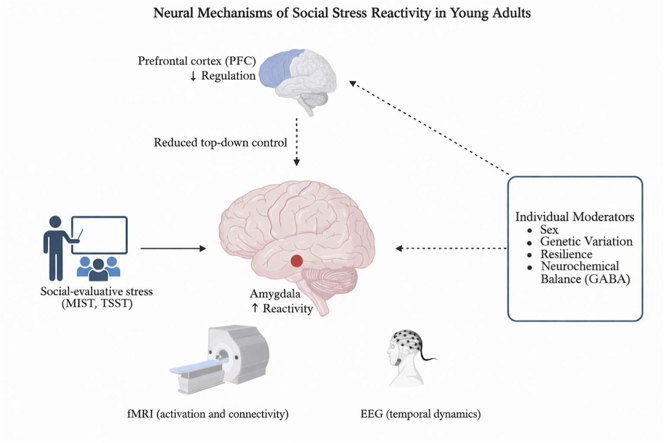

## 1 Introduction

Stress is an inescapable component of human life. It exerts profound effects on cognitive, emotional, and physiological functioning (Muscatell et al., 2022). Beyond its well-documented impact on mental health, chronic or intense stress can also contribute to adverse physical outcomes (Singh and Sachdev, 2019). Exposure to social stressors initiates coordinated neural responses involved in threat detection, emotional learning, and adaptive behavioral responses (Eisenberger, 2013; Hermans et al., 2014). Central to these processes is the amygdala, an almond-shaped structure within the medial temporal lobe that plays a critical role in emotional processing, fear learning, and the detection of socially relevant threats (AbuHasan et al., 2023; Salzman, 2025).

Importantly, the amygdala does not operate in isolation but interacts dynamically with cortical regions, particularly the prefrontal cortex (PFC), which plays a key role in integrating emotional and cognitive processes (Salzman and Fusi, 2010). The dorsolateral PFC (dlPFC) supports cognitive control and the regulation of impulsive responses, whereas the ventromedial PFC (vmPFC) contributes to emotional regulation by modulating fear and stress responses and exerting inhibitory control over the amygdala (Arnsten, 2009). Disruptions in amygdala-prefrontal circuitry have been implicated in heightened stress reactivity and increased vulnerability to anxiety, mood, and stress-related disorders (Arnsten, 2009; Berretz et al., 2021).

The involvement of the amygdala in stress processing is well established, as it plays a central role in detecting emotionally salient stimuli and coordinating autonomic and endocrine stress responses through its interactions with hypothalamic and brainstem regions (Herman et al., 2005; Ulrich-Lai and Herman, 2009). Prior studies have reported both heightened and attenuated amygdala responses, as well as variability in functional connectivity between the amygdala and prefrontal regulatory regions (Goodman et al., 2020; Orem et al., 2019). These inconsistencies may reflect differences in experimental paradigms, analytic approaches, developmental stage, and individual-level factors such as gender, cultural background, and stress resilience (Sudimac et al., 2022). Additionally, variability in amygdala reactivity may also arise from differences in underlying neurochemical regulation of stress responses.

Exposure to social stress activates the amygdala and initiates signaling to the hypothalamus, triggering the hypothalamic-pituitary-adrenal (HPA) axis and the release of cortisol (Barry et al., 2017). At the neurochemical level, amygdala responsivity is shaped by a balance between excitatory glutamatergic signaling, which facilitates threat learning and fear expression (Miranda et al., 2002; Guzmán-Ramos et al., 2012). Stress-related reductions in GABAergic transmission have been linked to heightened limbic activation and increased vulnerability to anxiety and mood disorders (Jie et al., 2018). These neurochemical processes provide an important mechanistic context for interpreting observed patterns of amygdala hyperactivation and disrupted amygdala-prefrontal connectivity in functional neuroimaging studies. Neuromodulatory systems, including norepinephrine and dopamine, further shape amygdala responsivity, particularly within the basolateral complex (Sharp, 2017), and may contribute to individual variability in stress-related amygdala-prefrontal dynamics observed in functional neuroimaging studies. Together, these neurochemical mechanisms help explain why amygdala responses to social stress vary across individuals and experimental contexts, motivating integrative synthesis of functional neuroimaging findings.

Functional magnetic resonance imaging (fMRI) has been particularly valuable in addressing these questions due to its high spatial resolution and sensitivity to subcortical structures such as the amygdala (Berretz et al., 2021). fMRI studies of social stress commonly employ validated experimental paradigms, including the Montreal Imaging Stress Task (MIST), Pavlovian fear conditioning, and the Trier Social Stress Test (TSST). The MIST induces psychosocial stress through time-pressured arithmetic and negative performance feedback (Dedovic et al., 2005), whereas Pavlovian fear conditioning captures anticipatory threat learning through repeated associations between neutral cues and aversive stimuli (Goodman et al., 2018). The TSST, by contrast, elicits strong social-evaluative stress via public speaking and mental arithmetic performed under observation (Allen et al., 2017). While these paradigms are often grouped under the umbrella of “social stress”, they may engage partially distinct psychological and neurobiological mechanisms, complicating direct comparison across studies.

Although this scoping review primarily focuses on fMRI studies due to their superior spatial resolution for assessing amygdala activity, electroencephalography (EEG) studies were included when they provided complementary temporal information on stress-related neural dynamics. Positron emission tomography (PET) studies were considered when they offered relevant neurochemical insights into stress-related processes. In both cases, these modalities were included primarily to contextualize and augment fMRI findings rather than serve as primary outcome evidence.

Young adulthood (approximately 17–35 years) represents a particularly relevant developmental window for examining these processes. This age range spans late adolescence through early adulthood, a transitional period during which functional integration between the amygdala and prefrontal cortex continues to mature into early adulthood, supported by ongoing development of fronto-limbic white matter pathways such as the uncinate fasciculus, which extends into the third decade of life (Olson et al., 2015). Consistent with this, amygdala-prefrontal circuitry undergoes progressive maturation across childhood and adolescence, enabling the gradual integration of emotional and cognitive control systems (Johnson et al., 2009; Roeske et al., 2025). Young adulthood is therefore recognized as a distinct developmental stage characterized by continued psychological and neurobiological maturation, even as changes become more gradual than in earlier life stages (National Academies of Sciences, Engineering, and Medicine, 2015). This period is associated with increased exposure to socially evaluative contexts during educational, occupational, and interpersonal transitions (Eisenberger, 2013). It is also characterized by the emergence of many mood and anxiety disorders during late adolescence and early adulthood (Solmi et al., 2022). Younger adolescents were not included in the present review to reduce developmental heterogeneity and because validated neuroimaging-adapted social stress paradigms such as the Montreal Imaging Stress Task (MIST) and the Trier Social Stress Test (TSST) have been primarily developed and most consistently applied in late adolescent and adult populations (Dedovic et al., 2005; Allen et al., 2017), with continued application in young adult and adult neuroimaging samples (e.g., Velozo et al., 2021; Erhart et al., 2024). Understanding how the amygdala responds to social stress during this stage may therefore provide critical insight into mechanisms of vulnerability and resilience.

To date, no synthesis has systematically examined how methodological and analytic variability shapes reported amygdala responses to social stress specifically within the young adult population. Accordingly, the present scoping review aims to synthesize fMRI studies examining amygdala responses to social stress in young adults. By comparing activation patterns and amygdala-prefrontal connectivity across paradigms and analytic approaches, this review seeks to clarify sources of inconsistency in the literature, identify methodological limitations, and highlight gaps that warrant future investigation. In doing so, this work aims to advance a more coherent understanding of the neural mechanisms underlying social stress and inform future research and intervention strategies targeting stress-related mental health outcomes in young adults.

## 2 Methods

### 2.1 Protocol and registration

This scoping review was conducted in accordance with the PRISMA-ScR guidelines (Tricco et al., 2018). Ethical approval was not required because the study synthesized previously published data and did not involve human participants.

### 2.2 Search strategy

A literature search was conducted in PubMed, ScienceDirect, and Google Scholar. Additional records were screened from BioRxiv to identify relevant preprints that were subsequently verified for peer-reviewed publication status when available. The goal was to identify eligible neuroimaging studies investigating amygdala-related responses to social stress in young adults, with fMRI studies constituting the primary focus. The search included human studies published from 2018 to 2024, and was limited to English-language publications.

The following Boolean logic-based search strategy was applied (with minor modifications across platforms):

(“amygdala” AND “social stress”) AND (“fMRI” OR “functional magnetic resonance imaging” OR “PET” OR “positron emission tomography” OR “EEG” OR “electroencephalography”) AND (“young adults”) AND (“MIST” OR “Montreal Imaging Stress Task” OR “TSST” OR “Trier Social Stress Test” OR “Pavlovian fear conditioning” OR “social judgment” OR “peer rejection” OR “social evaluation”).

Search terms included both keywords and MeSH terms when available. Additional records were identified by manually screening reference lists of included articles.

Although the search strategy included PET and EEG-related terms to ensure comprehensive coverage, fMRI studies constituted the primary focus of the review, with PET and EEG studies retained only when they provided complementary mechanistic or contextual insights relevant to amygdala-prefrontal stress regulation.

### 2.3 Eligibility criteria

Studies were selected based on predefined inclusion and exclusion criteria. Eligible studies were observational in nature, including cross-sectional and experimental designs, published between 2018 and 2024, conducted in human participants, and written in English. The target population comprised young adults aged 17 to 35 years, a developmental period characterized by continued heightened sensitivity to social stress (Eisenberger, 2013).

Functional magnetic resonance imaging (fMRI) studies constituted the primary inclusion criterion, given their capacity to directly assess amygdala activation and functional connectivity. Positron emission tomography (PET) and electroencephalography (EEG) studies were included only when they examined social stress-related processes relevant to amygdala function, such as neurochemical signaling or cortical regulatory dynamics, and were used to complement and contextualize fMRI findings rather than serve as primary outcome evidence.

Included studies were required to employ validated social stress paradigms designed to elicit social-evaluative threat, including the Montreal Imaging Stress Task (MIST), Pavlovian fear conditioning, the Trier Social Stress Test (TSST), or equivalent paradigms. For Pavlovian fear conditioning studies, only paradigms incorporating socially relevant stimuli (such as social evaluation cues, socially relevant threat stimuli, or interpersonal feedback contexts) were considered eligible.

Studies involving participants outside the 17–35 age range were excluded. The employment of alternative non-validated stress paradigms was also ruled out, to avoid introducing variability that could complicate data synthesis. Furthermore, studies involving individuals with diagnosed neurological, psychiatric, or neurodevelopmental disorders were excluded to avoid compounding effects on amygdala activity unrelated to social stress.

### 2.4 Study selection and data collection process

Search and identification of eligible studies were conducted by one reviewer, with a second reviewer consulted to resolve uncertainties and ensure consistency. All retrieved references were imported into Mendeley software, and duplicates were removed. Titles and abstracts were screened against the eligibility criteria, followed by full-text assessment of potentially relevant studies. Any uncertainties regarding study inclusion were resolved through discussion and cross-checking.

The following data were extracted from each included study: author(s) and year of publication; sample size; participant age range and reported demographics; stress paradigm details; imaging modality and acquisition parameters; preprocessing/analysis approach; and outcomes including amygdala activation and connectivity. For fMRI studies, data extraction also documented whether amygdala activity was assessed using region-of-interest (ROI) analyses or whole-brain approaches, as this distinction has implications for sensitivity and comparability across studies. The extracted data were stored in a Microsoft Excel spreadsheet.

### 2.5 Methodological quality and risk of bias assessment

Given the scoping nature of this review, assessment of risk of bias was conducted to characterize the methodological quality of included studies rather than to exclude studies or weight findings quantitatively. Risk of bias was evaluated for all included non-randomized studies using the Risk of Bias in Non-Randomized Studies of Interventions (ROBINS-I) tool (Sterne et al., 2016). Each study was assessed across the seven ROBINS-I domains: bias due to confounding; bias in classification of interventions; bias in selection of participants; bias due to deviations from intended interventions; bias due to missing data; bias in measurement of outcomes; and bias in selection of the reported result.

For each domain, risk of bias was judged as low, moderate, or serious according to ROBINS-I criteria, and an overall judgment was assigned to each study. Assessments were conducted by one reviewer and verified by a second reviewer to enhance consistency. The ROBINS-I signaling questions and a standardized assessment framework were applied throughout the evaluation process.

## 3 Results

### 3.1 Study selection and characteristics

**Fig. 1:**
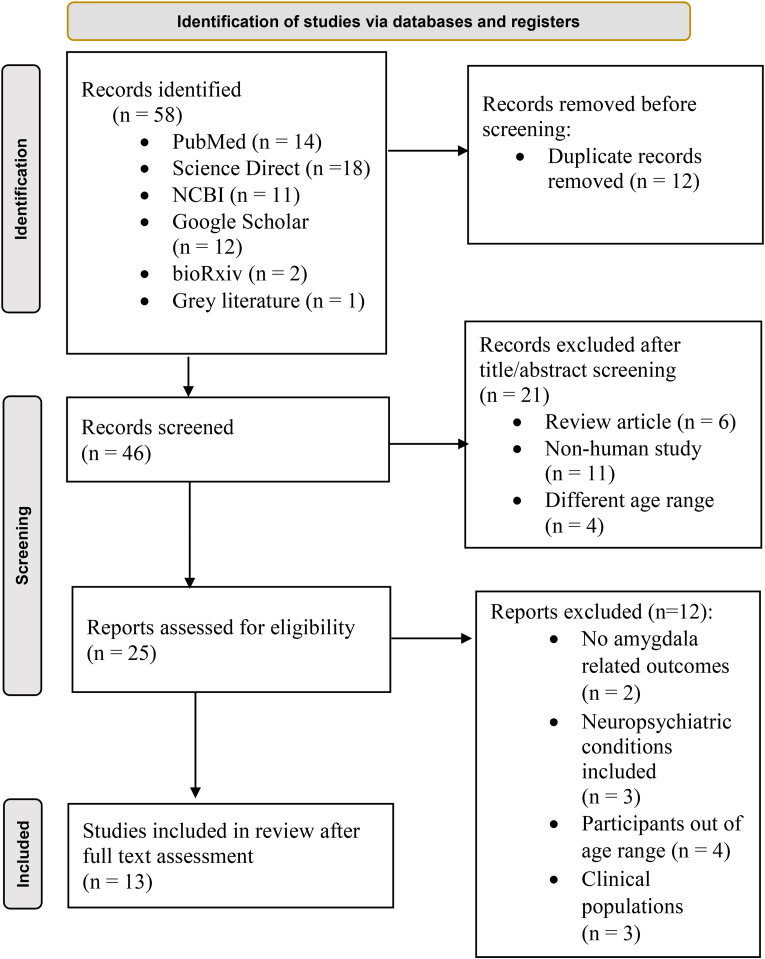
Detailed PRISMA-ScR flow diagram illustrating the selection process for studies included in the review.

Thirteen studies met the predefined eligibility criteria and were included in this scoping review. The study selection process is summarized in the PRISMA-ScR flow diagram (Fig. 1). A total of 58 records were initially identified across databases and reference list screening. After removal of 12 duplicates, 46 records underwent title and abstract screening, of which 21 were excluded for not meeting eligibility criteria. Full-text assessment was conducted for 25 articles, resulting in the inclusion of 13 studies that satisfied all selection criteria. Key methodological and participant characteristics were subsequently extracted and synthesized.

### 3.2 Demographics and paradigm details

**Fig. 2:**
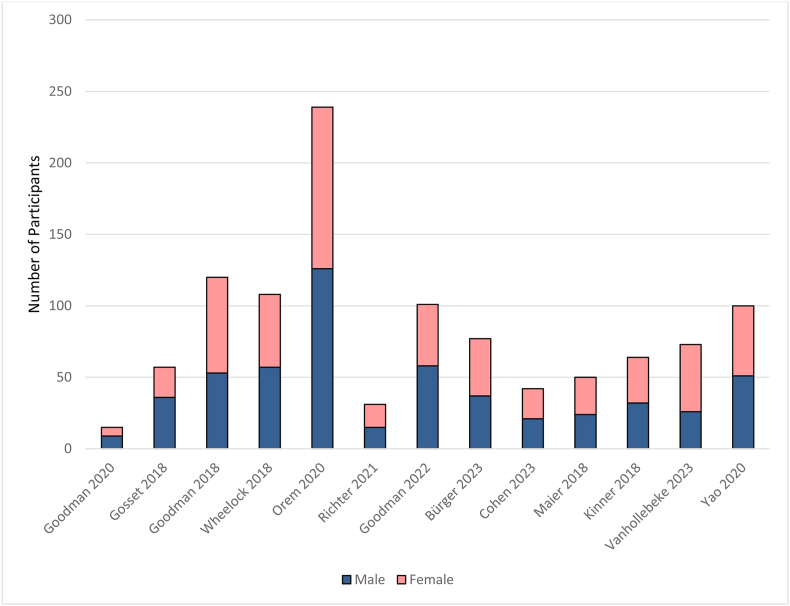
Sample size and gender distribution across included studies.

Across the 13 included studies, a total of 1077 healthy participants were reported in alignment with inclusion criteria. As summarized in Table 1, the reporting of age varied, some studies reported mean age and standard deviation (Goodman et al., 2020: M = 32.0, SD = 9.03), while others simply provided the age ranges (Gossett et al., 2018: 17-22). Additionally, several studies (including Richter et al., 2021), reported both the age range and mean age (18-31; M = 23.39, SD = 3.26). Gender distribution was reported in all studies and was generally balanced between males and females (Fig. 2). Across the included studies, a variety of validated social stress paradigms were employed. MIST was the most common, sometimes combined with Pavlovian fear conditioning (Goodman et al., 2018, 2022). Other paradigms included the TSST, the social judgment paradigm, and peer comparison tasks, which broaden the scope of social stressors represented in this review. Together, these paradigms enabled the induction of social-evaluative stress across diverse experimental contexts and facilitated the assessment of amygdala activity and its regulation under stress.

**Table 1:** Demographic and paradigm details of included studies on social stress and amygdala activity.

Author(s) & Year	Sample Size	Age Range | Mean (SD)	Gender Distribution	Social Stress Paradigm	Key Findings
Goodman et al. (2020) [23]	15	--- |32.00 (9.03)	9 M^a^, 6 F^b^	MIST	Reliable BOLD response across visits, it validates MIST as a reliable tool for studying stress responses; the dACC, dlPFC, insula, hippocampus showed activation during stress.
Gossett et al. (2018) [27]	57	17-22 |19.68	36 M, 21 F	MIST	The stress elicited by anticipation of fMRI may lead to acute elevations in cortisol prior to scanning, which may in turn disrupt the cortisol response to stress tasks performed during scanning.
Goodman et al. (2018) [10]	120	18-23 | 20.15	53 M, 67 F	MIST + Pavlovian fear conditioning	High stress-reactivity is linked to enhanced fear learning, higher SCR, disrupted emotion regulation; altered PFC and amygdala dynamics.
Wheelock et al. (2018) [20]	108	17-22 |18.88	57 M, 51 F	MIST	Stress decreased overall brain network efficiency; the vmPFC and hippocampus became less central in coordinating communication between brain regions, while the amygdala's role as a communication hub correlated with cortisol reactivity.
Orem et al. (2019) [11]	239	17-22 | ---	126 M, 113 F	MIST	Amygdala and PFC activity underlie individual differences in emotional response to stress; the vmPFC and dlPFC are positively associated with perceived stress and SCR.
Richter et al. (2021) [22]	31	18-31 | 23.39 (3.26)	15 M, 16 F	MIST	The interaction between genetics and environmental stressors in shaping brain responses to stress is highlighted.
Goodman et al. (2022) [21]	101	18-23 | ---	58 M, 43 F	MIST + Pavlovian fear conditioning	Acute stress can interfere with how the brain regulates emotions during threats. The PFC-amygdala connection plays a major role in how stress changes emotional reactions to threats.
Bürger et al. (2023) [12]	77	--- | 24.35 (3.4)	37 M, 40 F	MIST	Females exhibited stronger amygdala-frontal connectivity than males, higher prevalence rates of stress-related disorders in females.
Cohen et al. (2023) [24]	42	18-25 | 21.65 (0.1)	21 M, 21 F	MIST	Gender-based variations in neural mechanisms of stress, which could help explain sex differences in stress-related disorders.
Maier et al. (2019) [30]	50	--- | 24.54 (3.09)	24 M, 26 F	TSST^d^	Oxytocin attenuated amygdala, hippocampal, and ACC activation to chemosensory stress cues and reinstated ACC-FFA connectivity, reducing fear perception biases.
Kinner et al. (2018) [31]	64	18-40 | ---	32 M, 32 F	Fear conditioning	Cortisol enhanced right amygdala activation and fear recovery in men, but reduced amygdala activation in women, revealing sex-specific effects of stress hormones on relapse of fear.
Vanhollebeke et al. (2023) [29]	73	18-47 | 22.8 (5.73)	26 M, 47 F	Negative peer comparison task	Negative peer comparison reduced activity in self-reflection brain regions and altered their connectivity.
Yao et al. (2020) [32]	100	--- | 20.28 (0.305)	51 M, 49 F	Social judgment task	High pain sensitivity amplified early rejection responses, reflecting amygdala reactivity; later responses to unexpected rejection suggested amygdala-prefrontal regulation.

Note.

^a^ Male.

^b^ Female.

MIST = Montreal Imaging Stress Task; TSST = Trier Social Stress Test; SCR = Skin Conductance Response; fMRI = functional magnetic resonance imaging; dACC = dorsal anterior cingulate cortex; dlPFC = dorsolateral prefrontal cortex; BOLD = blood-oxygen-level-dependent signal; PFC = prefrontal cortex; vmPFC = ventromedial prefrontal cortex; FFA = fusiform face area.

### 3.3 Imaging techniques and preprocessing approaches

Neuroimaging techniques across the included studies primarily consisted of functional magnetic resonance imaging (fMRI; 11 studies), which formed the core evidence base of this review, alongside a smaller number of electroencephalography (EEG) studies (2 studies) that provided complementary temporal and regulatory insights. No eligible PET studies were identified.

Among the fMRI studies, most experiments were conducted using 3T scanners, with nine studies relying on Siemens platforms including the Allegra, Prisma, and Magnetom Trio models, which provide high spatial resolution suitable for detecting amygdala activation. Concerning acquisition protocols, most studies employed BOLD (blood-oxygen-level-dependent) echo-planar imaging sequences.

Preprocessing and analysis pipelines varied across studies. Three studies reported using AFNI for preprocessing and statistical analysis (Goodman et al., 2018, 2020; Orem et al., 2019), while Richter et al. (2021) used SPM8 for preprocessing and modeling. Additional analytic approaches were employed to examine network-level stress responses, including dynamic Granger causality analyses to assess effective connectivity (Wheelock et al., 2018). More recent studies adopted expanded analytic frameworks, such as resting-state functional connectivity analyses (Bürger et al., 2023) and multimodal integration of BOLD imaging with GABA magnetic resonance spectroscopy (Cohen et al., 2023).

Table 2 summarizes acquisition and preprocessing characteristics across studies. With respect to analytic strategy, fMRI studies varied in whether amygdala outcomes were derived from region-of-interest (ROI)-restricted analyses, whole-brain voxel-wise approaches, or mixed analytic frameworks combining whole-brain activation with ROI-based connectivity analyses (Table 3). Six studies used hypothesis-driven ROI-restricted analyses targeting predefined limbic structures, whereas four studies employed whole-brain or mixed approaches.

**Table 2:** Imaging techniques and preprocessing methods used in included fMRI studies.

Author(s) & Year	Imaging Technique
Goodman et al. (2020)	fMRI (3T Siemens Prisma, BOLD-EPI, analyzed with AFNI)
Gossett et al. (2018)	fMRI
Goodman et al. (2018)	fMRI (3T Siemens Allegra, BOLD EPI; analysis via AFNI)
Wheelock et al. (2018)	fMRI (3T Siemens Allegra, BOLD EPI); connectivity analysis via dynamic Granger causality)
Orem et al. (2019)	fMRI (BOLD), AFNI preprocessing
Richter et al. (2021)	fMRI (3T Siemens Magnetom TIM Trio), SPM8 preprocessing
Goodman et al. (2022)	fMRI (3T Siemens Allegra, BOLD)
Bürger et al. (2023)	fMRI (3T Siemens TIM Trio Scanner), resting-state functional connectivity (rsFC)
Cohen et al. (2023)	fMRI (3T Siemens Magnetom Prisma), BOLD + GABA MRS
Maier et al. (2019)	fMRI (3T Siemens, BOLD EPI, preprocessing and analysis via SPM12, GLM approach)
Kinner et al. (2018)	fMRI (3T Philips Achieva, T1 structural + T2 EPI, 2 mm voxels, SPM8 GLM analysis).
Vanhollebeke et al. (2023)	EEG (64-channel Waveguard cap, 512 Hz, ICA preprocessing; source-level power and connectivity analyses in BrainVision Analyzer).
Yao et al. (2020)	EEG (64-channel Brain Products, 1000 Hz; ICA preprocessing, ERP and time-frequency analysis of frontal-midline theta).

Note.

fMRI = functional magnetic resonance imaging; BOLD = blood-oxygen-level-dependent; EPI = echo-planar imaging; AFNI = Analysis of Functional NeuroImages; SPM = Statistical Parametric Mapping; rsFC = resting-state functional connectivity; GABA = gamma-aminobutyric acid; MRS = magnetic resonance spectroscopy; GLM = general linear model; EEG = electroencephalography; ICA = independent component analysis; ERP = event-related potential.

**Table 3:** Analytic approaches and ROI definitions used to assess amygdala activity across included fMRI studies.

Author & Year	Analytic Approach	Amygdala Assessment	atlas	ROI source	# ROIs
Goodman et al. (2020)	ROI-restricted	Amygdala activation examined within predefined anatomical ROI mask.	N/A	Anatomical ROI mask	Bilateral amygdala (2)
Gossett et al. (2018)	Not assessed	Amygdala not directly assessed; fMRI used as stress context.	N/A	N/A	N/A
Goodman et al. (2018)	ROI-restricted (hypothesis-driven)	Amygdala examined within anatomically defined ROI.	AFNI Talairach and Tournoux Atlas	Hypothesis-driven anatomical ROIs (PFC, amygdala, hippocampus, parahippocampal gyrus, insula, cingulate, inferior parietal lobule)	7 main anatomical regions
Wheelock et al. (2018)	Mixed (whole-brain + ROI-based)	Amygdala assessed as a network node using graph-theory metrics.	WFU PickAtlas (MNI space)	Activation-derived ROIs from prior MIST study (Wheelock et al., 2018) with 4-mm spherical ROIs	106 ROIs
Orem et al. (2019)	ROI-restricted voxel-wise	Amygdala assessed using voxel-wise activation with small-volume correction.	Automated Anatomical Labeling (AAL) atlas	ROI-restricted gray matter mask including PFC, amygdala, hippocampus, insula, and hypothalamus	N/A
Richter et al. (2021)	ROI-restricted	Amygdala-hippocampus activity assessed using predefined spherical ROIs with FWE-corrected SVC.	Neurosynth-derived coordinates (MNI space)	Small-volume ROIs defined as 4-mm spheres around meta-analytic coordinates (ACC and amygdala/hippocampus)	4 ROIs
Goodman et al. (2022)	Mixed (whole-brain, functional ROI, effective connectivity)	Amygdala assessed as a functionally defined ROI node in directional effective connectivity analyses.	Functionally defined coordinates (Talairach space)	8-mm spherical ROIs centered on activation peaks identified in a prior fMRI analysis	21 ROIs
Bürger et al. (2023)	ROI-based	Bilateral amygdala defined as seed ROIs for resting-state functional connectivity with frontal regions.	MNI coordinate-based ROIs	6-mm spherical ROIs centered on MNI coordinates derived from prior literature	6 ROIs
Cohen et al. (2023)	Mixed (whole-brain activation, ROI-based connectivity)	Amygdala examined using whole-brain contrasts and seed-based connectivity analyses.	Anatomical masks (custom masks/MNI-based)	Seed regions defined using anatomical masks created by the Core Morphology Group at Martinos Center for Biomedical Imaging	6 seed ROIs
Maier et al. (2019)	Mixed (whole-brain, ROI-based, connectivity)	Amygdala activation detected in whole-brain analyses and further examined using hypothesis-driven limbic ROI extraction.	Functional cluster-derived spheres (MNI coordinates)	ROIs defined as 4-mm radius spheres centered on peak activation coordinates from the BOLD analysis	∼3–4 seed ROIs depending on analysis (ACC, amygdala, hippocampus)
Kinner et al. (2018)	ROI-restricted	Amygdala assessed using Harvard-Oxford anatomical ROI; FWE-corrected SVC.	Harvard–Oxford Cortical and Subcortical Structural Atlases (probability threshold = 0.25)	Anatomical masks for fear circuitry regions; additional spherical ROIs from meta-analytic peak coordinates (vmPFC, dACC)	7 ROIs

Note.

This table summarizes the analytic strategies used to assess amygdala-related outcomes across included functional magnetic resonance imaging (fMRI) studies. ROI-restricted refers to analyses confined to predefined anatomical or functional regions of interest, typically using small-volume correction. Whole-brain refers to voxel-wise analyses conducted across the entire brain without a priori regional restriction. Mixed approaches combined whole-brain activation analyses with subsequent ROI-based signal extraction, connectivity, or network-level analyses. The studies also varied in the granularity of ROI definition, ranging from coarse anatomical masks (e.g., atlas-based amygdala regions) to more granular parcellations using coordinate-derived spherical ROIs or large network-based ROI sets. EEG studies are not included in this classification because EEG does not allow direct spatial localization of subcortical structures such as the amygdala.

In addition to analytic strategy, the studies differed in the granularity of ROI definition and atlas selection. Some investigations used relatively coarse anatomical masks derived from standard atlases (such as Harvard-Oxford or AAL), treating the amygdala as a single anatomical region. Others used more granular approaches, including coordinate-based spherical ROIs or large network parcellations containing dozens of nodes across the brain. These methodological differences in ROI specification may influence sensitivity to localized amygdala responses and contribute to variability across reported findings.

While all studies shared the common aim of probing amygdala-related stress responses, variation in scanner platforms, preprocessing pipelines, ROI definitions, and analytic strategies introduces methodological heterogeneity that may affect comparability across studies.

### 3.4 fMRI findings

The following section summarizes findings from the 11 fMRI studies included in this review, focusing first on patterns of amygdala activation during social stress and then on its functional connectivity with other brain regions.

i. Amygdala Activation Patterns

During the Montreal Imaging Stress Task (MIST), several studies reported increased amygdala activation in response to socially evaluative performance, consistent with its role in stress reactivity. For example, Orem et al. (2019) reported increased right amygdala activation during MIST-induced social evaluative stress, and observed a positive correlation between right amygdala activity and skin conductance responses (SCR), supporting the link between neural and autonomic arousal.

Environmental and genetic factors also influenced amygdala responses. In a stress paradigm involving socially evaluative threat, Richter et al. (2021) observed heightened amygdala reactivity in individuals carrying the FKBP5 A/A genotype, particularly among participants exposed to urban living environments, suggesting an interaction between genetic vulnerability and environmental stress exposure.

Some studies did not report significant amygdala activation (Goodman et al., 2020; Gossett et al., 2018), which may reflect differences in task design, analytic strategy, or imaging methodology rather than the absence of stress-related amygdala engagement.

Methodological differences in analytic approach likely contributed to this variability. Studies using hypothesis-driven ROI-restricted analyses were generally more sensitive to detecting amygdala activation than those relying solely on whole-brain voxel-wise analyses (Table 3). In addition, variation in ROI definitions and atlas selection across studies may have influenced the spatial specificity and detectability of amygdala responses.

Taken together, these findings suggest that while amygdala activation during social stress is commonly observed, its detection depends strongly on task design and analytic methodology.

ii. Functional Connectivity

Beyond regional activation patterns, several studies discussed the amygdala's functional interactions with other brain regions under social stress. A consistent finding was the role of the PFC in modulating amygdala activity, specifically its subdivisions: the medial (mPFC), the dorsomedial (dmPFC), and the dorsolateral (dlPFC). Weaker functional connectivity was reported between the amygdala and prefrontal regions during stress-inducing tasks, implying impaired top-down emotional regulation. People with lower trait resilience showed weaker amygdala-dlPFC connectivity (Wheelock et al., 2018), while decreased amygdala-mPFC connectivity was seen during MIST performance (Orem et al., 2019). Chronically stressed individuals also showed altered baseline amygdala-frontal connectivity (Bürger et al., 2023). Neurochemical modulation played a role as well: low GABA concentrations were linked to weaker functional regulation and elevated amygdala reactivity (Cohen et al., 2023). Effective connectivity analyses confirmed disrupted top-down control from the PFC to the amygdala under stress (Goodman et al., 2022). These findings indicate that amygdala responses are closely linked to interactions with regulatory regions.

### 3.5 EEG findings

As a complementary component of this scoping review, EEG findings are summarized to contextualize fMRI-based observations by highlighting temporal and cortical dynamics associated with social stress. Unlike fMRI, which captures spatially localized activation patterns, EEG allows for precise tracking of oscillatory activity and connectivity changes over time. The following subsections summarize findings related to stress-induced oscillatory responses and source-level connectivity.

i. Neural Oscillatory Responses

EEG studies revealed that psychosocial stress alters oscillatory brain activity, particularly within low-frequency bands associated with threat processing and regulatory control. In a social rejection paradigm, Yao et al. (2020) reported that individuals with high pain sensitivity (HPS) exhibited stronger early theta and delta oscillatory responses to rejection compared with acceptance. These early responses likely reflect rapid detection of socially threatening stimuli. Later in the task, both high and low sensitivity groups demonstrated stronger theta and delta activity when rejection occurred unexpectedly, indicating that violations of social expectations intensified neural responses to negative feedback.

In a peer-feedback paradigm, Vanhollebeke et al. (2023) observed increased alpha oscillatory activity following negative peer evaluation. Source-level analyses localized these changes to regions involved in self-referential processing, including the precuneus and posterior cingulate cortex. These findings suggest that social evaluative stress may transiently alter self-related processing and attentional dynamics.

Together, these results indicate that socially evaluative stress modulates low-frequency oscillatory dynamics, with individual traits such as pain sensitivity influencing the magnitude of neural responses.

ii. Connectivity and Source-Level Dynamics

EEG studies also showed that social stress changes how different brain regions work together. During negative peer feedback, the left and right precuneus became more strongly connected (Vanhollebeke et al., 2023), suggesting that stress disrupts normal self-reflection processes. Additionally, personality traits influenced these responses: participants with high pain sensitivity showed stronger early reactions to rejection, while later brain responses were especially strong when rejection was unexpected, no matter the group (Yao et al., 2020). These results indicate that stress not only changes local brain rhythms but also alters communication between regions. While EEG cannot directly measure the amygdala, the patterns it reveals support fMRI findings that stress weakens top-down control and increases sensitivity to social threats.

### 3.6 Individual and demographic factors

In addition to task and connectivity-based findings, several studies showed that individual factors influence amygdala responses to social stress. Women displayed higher amygdala activation than men, with sex-specific connectivity patterns in amygdala-prefrontal circuits (Bürger et al., 2023; Cohen et al., 2023). Genetic variation (like FKBP5 A/A genotype) and environmental stress exposure were linked to heightened amygdala reactivity (Richter et al., 2021). Higher resilience was associated with stronger amygdala-dlPFC connectivity, suggesting better regulatory control under stress (Wheelock et al., 2018).

### 3.7 Methodological quality and risk of bias

Given the scoping nature of this review, risk of bias assessment was used to characterize methodological quality rather than to exclude studies or weight findings quantitatively. Of the thirteen selected studies, most were judged to have a moderate overall risk of bias. The domain-level judgment for each study is summarized in Table 4, where multiple studies consistently exhibited moderate concerns about confounding and selection of participants; these concerns may reflect baseline group differences or volunteer effects. Nonetheless, the consistent low-risk ratings for the classification of interventions and outcome measurements strengthen confidence that the measured neural variables likely reflect genuine stress-related effects. A few studies reported notable dropouts or incomplete outcome data but did not detail how these data gaps were handled; consequently, several studies were rated as having a moderate risk of bias in this area. There was minimal concern for intervention deviation, and most studies indicated low risk, showing good fidelity to the interventions. Two studies (Kinner et al., 2018; Vanhollebeke et al., 2023) were rated as low overall risk, reflecting rigorous design and reporting. Importantly, no studies were rated at critical risk of bias.

**Table 4:** Summary of ROBINS-I domain-level risk of bias judgments for included studies.

Author & Year	1	2	3	4	5	6	7	Overall Risk of Bias
Goodman et al. (2020)	moderate	low	moderate	low	moderate	low	low	moderate
Gossett et al. (2018)	moderate	low	moderate	moderate	moderate	low	moderate	moderate
Goodman et al. (2018)	moderate	low	moderate	low	moderate	low	moderate	moderate
Wheelock et al. (2018)	moderate	low	moderate	low	moderate	low	moderate	moderate
Orem et al. (2019)	moderate	low	moderate	low	moderate	low	moderate	moderate
Richter et al. (2021)	moderate	low	moderate	low	moderate	moderate	moderate	moderate
Goodman et al. (2022)	moderate	low	moderate	low	moderate	moderate	moderate	moderate
Bürger et al. (2023)	moderate	low	moderate	low	moderate	low	moderate	moderate
Cohen et al. (2023)	moderate	low	moderate	low	moderate	low	moderate	moderate
Maier et al. (2019)	moderate	low	low	low	low	moderate	low	moderate
Kinner et al. (2018)	moderate	low	low	low	low	low	low	low
Vanhollebeke et al. (2023)	low	low	low	low	low	moderate	low	low
Yao et al. (2020)	moderate	low	low	moderate	low	moderate	moderate	moderate

Note.

Risk of bias was assessed using the ROBINS-I tool.

1 = Bias due to confounding; 2 = Bias in classification of interventions; 3 = Bias in selection of participants; 4 = Bias due to deviations from intended interventions; 5 = Bias due to missing data; 6 = Bias in measurement of outcomes; 7 = Bias in selection of reported results.

Low = low risk of bias; Moderate = moderate risk of bias.

## 4 Discussion

### 4.1 Summary of main findings

Across the thirteen studies included in this scoping review, social stress was commonly associated with increased amygdala reactivity and altered functional connectivity between the amygdala and prefrontal regulatory regions. These neural patterns were not uniform across studies, reflecting the influence of individual differences, task design, and analytic approaches. Together, the findings suggest that social stress in young adults is characterized by heightened limbic responsivity alongside reduced top-down regulatory control, underscoring the complexity and heterogeneity of neural stress responses.

### 4.2 Interpretation and implications

i. Amygdala Reactivity and the Breakdown of Regulatory Control

A central pattern observed across the reviewed literature is heightened amygdala activation during socially evaluative stressors, such as judgment, performance pressure, or social exclusion. Importantly, this heightened reactivity appears to be closely linked to the integrity of prefrontal regulatory mechanisms. Several studies reported weakened functional connectivity between the amygdala and prefrontal regions during stress, suggesting compromised top-down modulation of emotional responses. Such disruptions were associated with greater emotional reactivity and potentially reduced stress coping capacity. Complementary EEG findings further supported this interpretation by revealing early delta and theta oscillatory responses to social rejection, reflecting rapid threat detection processes that parallel limbic hyperreactivity observed in fMRI.

ii. Individual Differences in Neural Stress Responses

**Fig. 3:**
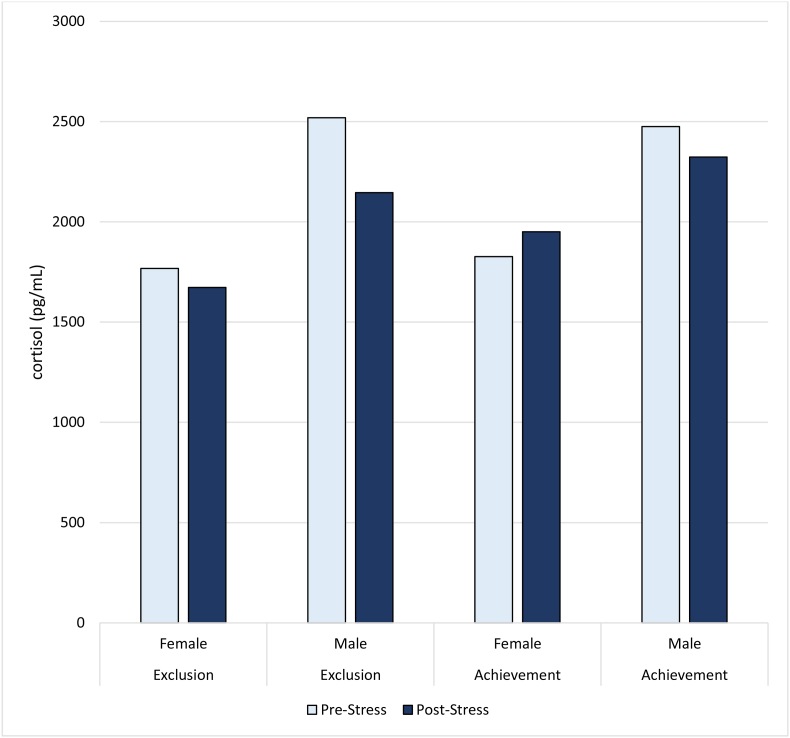
Cortisol levels before and after stress exposure by sex and stress type (social exclusion vs. achievement stress). Data extracted from Bürger et al. (2023) and visualized by the author.

The reviewed studies consistently demonstrated that neural responses to social stress vary substantially across individuals. Sex-related differences emerged across several investigations, with women showing greater activation in limbic and ventromedial prefrontal regions (Cohen et al., 2023), whereas men more frequently engaged dorsolateral prefrontal and anterior cingulate networks, suggesting differential reliance on emotional versus cognitive regulatory strategies. Physiological responses reflected these neural patterns; males exhibited higher pre- and post-stress cortisol levels than females, especially in social exclusion stress (Bürger et al., 2023) as shown in Fig. 3. In addition, genetic factors, trait anxiety, and resilience significantly influenced amygdala reactivity and amygdala-prefrontal connectivity, highlighting the importance of integrating biological and psychological factors when modeling stress vulnerability. Pharmacological findings supported these effects, as cortisol increased fear reinstatement in men, whereas oxytocin reduced stress-related biases (Maier et al., 2019).

iii. Neurochemical Modulation and The Role of GABA

Neurochemical evidence provided further insight into the mechanisms underlying stress-related neural dysregulation. Studies incorporating magnetic resonance spectroscopy revealed that reduced GABA concentrations in prefrontal and cingulate regions were associated with diminished functional connectivity to the amygdala and heightened emotional reactivity during stress. Conversely, higher prefrontal GABA levels were linked to more effective regulatory control and improved task performance under stress. These findings suggest that inhibitory neurotransmission plays a key role in shaping amygdala-prefrontal interactions during social stress. Other neuromodulators, including norepinephrine, dopamine, and oxytocin, were also implicated, underscoring the multifaceted neurochemical regulation of stress responses.

iv. Developmental Perspective on Amygdala-PFC Maturation

Young adulthood represents a developmental stage during which prefrontal regulatory systems continue to refine following earlier maturation of limbic structures. Developmental research indicates that amygdala-prefrontal circuitry undergoes progressive maturation across childhood and adolescence and continues to stabilize into early adulthood (Roeske et al., 2025). Within this broader developmental trajectory, the young adult period represents a stage in which regulatory networks are largely established but may remain sensitive to socially evaluative stressors. The reviewed findings support this developmental framework, suggesting robust amygdala reactivity and comparatively weaker prefrontal modulation during social stress in individuals aged 17–35. This developmental imbalance may contribute to increased vulnerability to anxiety and mood disorders during this life stage and highlights the importance of early interventions aimed at strengthening emotion regulation capacities.

v. Rationale for using fMRI as the Primary Methodological Approach

Functional magnetic resonance imaging served as the primary methodological foundation of the reviewed literature because of its high spatial resolution and ability to reliably assess subcortical structures such as the amygdala. While PET and EEG offer valuable molecular and temporal information, respectively, they present limitations for isolating rapid, task-evoked amygdala responses in healthy young populations. Nonetheless, EEG findings in this review provided complementary evidence of rapid oscillatory dynamics associated with threat detection and regulatory processes, supporting and extending fMRI-based observations.

vi. Neuroimaging Considerations and Broader Implications

The reviewed studies highlight both the strengths and limitations of current neuroimaging approaches to social stress. Task-based fMRI enables real-time assessment of stress responses but relies on indirect measures of neural activity. Emerging multimodal approaches integrating fMRI with MRS or resting-state analyses have enhanced understanding of neurochemical and network-level mechanisms underlying stress reactivity.

An additional methodological consideration concerns the reliability of functional connectivity estimates, particularly for subcortical structures such as the amygdala. Functional connectivity measures can be sensitive to scan duration, preprocessing strategies, and signal-to-noise characteristics, and subcortical regions often require larger amounts of data to produce stable connectivity estimates. Prior work has shown that reliability of functional connectivity can be relatively low for short scanning sessions and improves with longer acquisition times (Noble et al., 2017). Variability in scan length and analytic pipelines across studies may therefore contribute to differences in reported amygdala-prefrontal connectivity patterns.

Although PET studies were absent from the eligible literature, future work combining PET with fMRI and EEG may offer a more comprehensive account of molecular, functional, and temporal dynamics involved in stress processing.

vii. Comparison with Previous Literature

The findings of this review align with previous work by Berretz et al. (2021) and Muscatell et al. (2022), confirming consistent activation of the amygdala, prefrontal cortex, and insula during social stress. However, this review extends earlier work by emphasizing individual differences in activation patterns, functional connectivity, and neurochemical modulation. Recent evidence by Liu et al. (2022) supports this personalized perspective, showing that resting-state connectivity can predict stress responses. Rather than viewing stress responses as uniform, the evidence supports a personalized framework in which stress-related neural dynamics vary across individuals based on biological, psychological, and contextual factors.

viii. Methodological Strengths and Limitations

This scoping review offers a focused examination of social stress-related neural mechanisms in young adults, integrating activation, connectivity, and neurochemical findings. However, variability in study design, analytic strategies, and sample characteristics limits direct comparability across studies and contributes to heterogeneity in reported amygdala findings. The relatively small number of eligible studies and the exclusion of non-English publications may also constrain generalizability. Despite these limitations, the review provides a structured synthesis of current evidence and identifies key directions for future research.

## 5 Recommendations for future research

Future investigations should prioritize larger and more diverse samples, longitudinal designs, and greater methodological standardization to enhance comparability across studies. Increased use of multimodal neuroimaging approaches, including combined fMRI, MRS, EEG, and where feasible PET, would enable more comprehensive characterization of stress-related neural mechanisms. Additionally, greater attention to individual-level moderators, such as endocrine status, genetic variation, and emotion regulation strategies, will be essential for developing personalized models of stress vulnerability and resilience.

## 6 Conclusion

This scoping review synthesized current neuroimaging evidence on the neural correlates of social stress in young adults, with a particular focus on amygdala activity and its functional integration with prefrontal regulatory systems. Across the included studies, socially evaluative stressors were commonly associated with heightened amygdala reactivity and altered amygdala-prefrontal connectivity, supporting the view that social stress engages both affective and regulatory neural circuits during a critical developmental period.

Beyond identifying shared neural patterns, this review highlights substantial heterogeneity in stress-related brain responses, shaped by individual, biological, and methodological factors. Variability related to sex, genetic susceptibility, neurochemical balance, and psychological resilience suggests that social stress cannot be adequately understood through a single, uniform neural model. Instead, the findings point toward a more individualized neurobiological framework in which vulnerability and resilience emerge from dynamic interactions between limbic reactivity and prefrontal control systems.

Importantly, this synthesis clarifies several directions for future research. First, it underscores the need for greater methodological consistency across social stress paradigms, imaging protocols, and analytic strategies to improve comparability and reproducibility. Second, it highlights the value of multimodal neuroimaging approaches that integrate the spatial precision of fMRI with the temporal sensitivity of EEG and the neurochemical specificity of MRS or PET, where feasible. Such integrative designs may enable a more comprehensive understanding of how molecular, network-level, and temporal processes jointly shape stress responses.

From a broader translational perspective, characterizing patterns of amygdala-prefrontal dysregulation may help guide future efforts to identify neural markers associated with stress sensitivity during young adulthood. While clinical application remains premature, this body of work provides a conceptual foundation for developing targeted prevention and resilience-based strategies tailored to individual neurobiological profiles. By mapping the current landscape of evidence and identifying key gaps, this scoping review contributes to a more nuanced and forward-looking understanding of the neurobiology of social stress and sets the stage for more precise, personalized research in the years ahead.

## Declaration of generative AI and AI-assisted technologies in the manuscript preparation process

During the preparation of this manuscript, the authors used ChatGPT (OpenAI) to assist with language editing and generation of a high-resolution graphical abstract based on an initial conceptual design. BioRender was used at the conceptualization stage to explore visual layout; however, the final figure was independently generated and finalized outside the BioRender platform. The authors reviewed and edited all content and take full responsibility for the accuracy and integrity of the published work.

## Funding

This research did not receive any specific grant from funding agencies in the public, commercial, or not-for-profit sectors.

## CRediT authorship contribution statement

**Maribelle Nassar:** Conceptualization, Data curation, Formal analysis, Investigation, Methodology, Writing – original draft, Writing – review & editing. **Hayat Harati:** Methodology, Validation, Visualization. **Elias Estephan:** Formal analysis, Methodology, Validation, Visualization, Writing – review & editing. **Elie Al Ahmar:** Formal analysis, Methodology, Validation, Visualization, Writing – review & editing. **Lea Saab:** Conceptualization, Formal analysis, Investigation, Methodology, Project administration, Supervision, Validation, Visualization, Writing – review & editing. **Mariebelle Saab:** Conceptualization, Formal analysis, Investigation, Methodology, Project administration, Supervision, Validation, Visualization, Writing – review & editing.

## Declaration of competing interest

The authors declare that they have no known competing financial interests or personal relationships that could have appeared to influence the work reported in this paper.

## Data Availability

Data will be made available on request.
